# Phylogenetic placement of two taxonomically controversial South American spleenworts with unusual morphology: *Asplenium brasiliense* Sw. and *Asplenium douglasii* Hook. & Grev. (Aspleniaceae)

**DOI:** 10.3897/phytokeys.278.198424

**Published:** 2026-07-24

**Authors:** Jing-Shuai Zhang, Ming-Hui Du, Li-Bing Zhang, Ke-Wang Xu

**Affiliations:** 1 Co-Innovation Center for Sustainable Forestry in Southern China, College of Life Science, Nanjing Forestry University, Nanjing 210037, China Co-Innovation Center for Sustainable Forestry in Southern China, College of Life Science, Nanjing Forestry University Nanjing China; 2 Missouri Botanical Garden, 4344 Shaw Blvd, St. Louis, MO 63110, USA Missouri Botanical Garden St. Louis United States of America

**Keywords:** *
Antigramma
*, fern phylogeny, *
Hemidictyum
*, *
Phyllitis
*, *
Scolopendrium
*, South American fern flora

## Abstract

The genus *Asplenium* is known for its high morphological diversity and complex evolutionary history. Two South American species, *Asplenium brasiliense* and *A. douglasii*, possess unusual morphological traits, including simple fronds, anastomosing venation, and paired indusia opening toward each other, which have historically led to their assignment to several satellite genera, such as *Antigramma*, *Hemidictyum*, *Phyllitis*, and *Scolopendrium*. However, their phylogenetic positions have never been rigorously tested using molecular data. In this study, four chloroplast markers were employed to infer the phylogenetic placement of these two enigmatic species. The phylogenetic analyses robustly resolved both species as members of the *Asplenium schaffneria* clade, where they form a strongly supported monophyletic group that is sister to the *A. varians* subclade. This study provides the first molecular phylogenetic evidence clarifying the phylogenetic relationships of *A. douglasii* and *A. brasiliense*, demonstrating that their morphological peculiarities represent specialized evolutionary derivations within *Asplenium* s.l. rather than providing justification for generic segregation.

## Introduction

*Asplenium* L. (Aspleniaceae) is the most species-rich and widely distributed genus of ferns, comprising approximately 700 species worldwide (Kramer and Viane 1990; Schneider et al. 2004; PPG I 2016). Frequent interspecific hybridization and polyploidization events have generated a complex reticulate evolutionary history (Lovis 1978; Walker 1979; Werth et al. 1985; Pinter et al. 2002; Dyer et al. 2012; Heo et al. 2022; Liang and Zhang 2024). Concurrently, adaptation to diverse ecological environments has driven the evolution of markedly distinct “survival morphologies” in ferns (Creese et al. 2011). Together, these complex evolutionary processes and ecological factors have resulted in the emergence of numerous morphologically distinctive lineages within *Asplenium*. For example, *Ceterach* Willd. (the spleenwort genus) has evolved thick leaves densely covered with scales on the abaxial surface as an adaptation to arid environments; *Neottopteris* J. Sm. (the bird’s-nest fern genus) has developed large, nest-like fronds that efficiently collect leaf litter and moisture. These striking morphological divergences have historically led taxonomists to recognize multiple satellite genera within Aspleniaceae. It was not until Xu et al. (2020) analyzed more than 1,000 samples representing approximately 420 species worldwide using six plastid markers that *Asplenium* s.l. was demonstrated to be monophyletic, with traditionally recognized satellite genera such as *Ceterach* and *Neottopteris* nested within it.

Among the taxa with uncertain phylogenetic placement, two South American species (*Asplenium brasiliense* Sw. and *A. douglasii* Hook. & Grev.) have particularly unusual taxonomic histories. Both species were originally described within *Asplenium* (Swartz 1817; Hooker and Greville 1829). However, based on their simple frond morphology and distinctive venation patterns, Presl (1836) transferred them to the genus *Hemidictyum* and later to the newly established genus *Antigramma* C. Presl (Presl 1836; Hooker and Bauer 1842). Subsequent studies incorporating stelar anatomy and spore ornamentation further supported the recognition of *Antigramma* as an independent genus (Hayata 1928; Sylvestre and Windisch 2002). As a result, these two species have long been treated under the generic name *Antigramma* in various taxonomic treatments (Moore 1857; Kuntze 1891; Hieronymus 1908). Their currently accepted names, *A. brasiliense* and *A. douglasii*, reflect a return to their original generic placement following the broader circumscription of *Asplenium* (e.g., Sylvestre and Windisch 2002; Xu et al. 2020). Despite this taxonomic history, the phylogenetic positions of these two species within *Asplenium* have never been rigorously tested using molecular data.

The present study aimed to (1) determine the precise phylogenetic positions of *A. brasiliense* and *A. douglasii* using four chloroplast markers, (2) elucidate the phylogenetic relationships among these two species and their closely related congeners, and (3) explore the morphological similarities between them and their related species.

## Materials and methods

### DNA extraction, PCR amplification, and sequencing

Material of *Asplenium brasiliense* and *A. douglasii* was obtained from the herbarium of the Missouri Botanical Garden (MO). Total genomic DNA was extracted from silica-gel-dried leaf material using a Plant Genomic DNA Kit (Tiangen Biotech, Beijing, China), following the manufacturer’s protocol. Four chloroplast markers were amplified: the *atpB* gene, the *rbcL* gene, the *rps4* and *rps4–trnS* regions, and the *trnL* and *trnL–trnF* regions, using primers described by Xu et al. (2018). Each polymerase chain reaction (PCR) was performed in a total volume of 20 μL containing 7 μL of 2× PCR Master Mix, 10 μL of ddH_2_O, 0.5 μL of each forward and reverse primer (10 μM), and 2 μL of DNA template. The PCR amplification program consisted of an initial denaturation at 95 °C for 5 min, followed by 40 cycles of denaturation at 95 °C for 45 s, annealing at 50 °C for 30 s, and extension at 72 °C for 1 min, with a final extension at 72 °C for 5 min. The PCR products were purified using TIANquick mini purification kits (TIANGEN, Beijing, China) and sequenced by Sangon Biotech Co., Ltd. (Shanghai, China).

### Sequence alignment and phylogenetic analysis

Newly generated raw sequences were assembled and edited using Sequencher v4.14 (Ann Arbor, Michigan, USA). Using the BLASTN program (Altschul et al. 1990), the newly generated sequences of *A. brasiliense* and *A. douglasii* were compared with those deposited in GenBank. The BLAST searches suggested an affiliation of both species with the *A. varians* subclade of the *A. schaffneria* clade of Xu et al. (2020). Therefore, all available *atpB*, *rbcL*, *rps4* and *rps4–trnS*, and *trnL* and *trnL–trnF* sequences of members of the *A. schaffneria* clade were downloaded. This initial dataset was reduced to one individual per species, prioritizing accessions with the most complete sequence fragments. Sequences from representative species of the remaining *Asplenium* clades and subclades retrieved by Xu et al. (2020) were also downloaded.

All sequences were initially aligned using MAFFT v7.480 (Tokyo, Japan) and subsequently adjusted manually in BioEdit v7.5.5 (Wooster, Ohio, USA) to ensure the accurate alignment of insertion–deletion events (indels). The final concatenated matrix had a total aligned length of 3,599 base pairs (bp) and comprised 115 samples representing 85 species. The resulting matrix was used for maximum likelihood (ML) phylogenetic analyses conducted on the CIPRES Science Gateway using RAxML-HPC2 on ACCESS v8.2.10 (San Diego, California, USA). Bayesian inference (BI) analyses were performed on the CIPRES platform using MrBayes v3.2.7a (Stockholm, Sweden). For the ML analyses, 1,000 bootstrap replicates were conducted to assess nodal support. For the BI analyses, two independent runs, each with four Markov chains, were performed for 1 million generations, with sampling every 1,000 generations. The first 25% of samples were discarded as burn-in, and convergence was confirmed by an average standard deviation of split frequencies < 0.01. The resulting phylogenetic trees were visualized using FigTree v1.4.3 (Rambaut 2016).

### Morphological and spore micromorphological analyses

To investigate the evolutionary patterns of morphological characters within the *A. varians* subclade, seven morphologically informative characters within *Asplenium* were selected based on previous studies of morphological diversity in the genus (e.g., Francis 1855; Monod 1976; Casas and Garmendia 1981; Sylvestre and Windisch 2002; Klopper and Crouch 2012; Brownsey and Perrie 2014; Ganem 2018; Xu et al. 2021; Lubienski 2022), together with a comprehensive examination of herbarium specimens available through online databases such as GBIF (https://www.gbif.org), the Pteridophyte Collections Consortium (https://www.pteridoportal.org), and Tropicos (https://www.tropicos.org). The selected characters included (1) degree of frond division, (2) overall frond shape, (3) ratio of petiole length to lamina length, (4) venation type, (5) sorus morphology, (6) relative length of the sorus with respect to the vein, and (7) presence or absence of auriculate lobes or short basal pinnae at the base of the frond. These characters were discretized according to diagnostic states commonly used in the taxonomic literature and identification keys and were coded as multistate characters with states ranging from 0 to 2. The coded morphological characters for all taxa were compiled into a morphological matrix, which was used for subsequent comparison and discussion in conjunction with the phylogenetic results.

For observations of spore morphology, two individuals per species were selected, and unopened sporangia were used to ensure that the spores were intact and had not been released. The unopened sporangia were mounted on specimen stubs using double-sided adhesive tape and then manually ruptured to release the spores. Spore morphology was examined using scanning electron microscopy (SEM), and micrographs of the perispore surface were obtained.

## Results

### Placement of *A. brasiliense* and *A. douglasii*

Phylogenetic analyses were conducted using ML and BI. The alignment used for the phylogenetic analyses included four chloroplast markers (*atpB*, *rbcL*, *rps4* and *rps4–trnS*, and *trnL* and *trnL–trnF*) and was 3,599 bp in length, of which 2,007 sites were constant, 1,107 were parsimony-informative, and 485 were variable but parsimony-uninformative. A total of seven sequences for the target species (*Asplenium brasiliense* and *A. douglasii*) were newly generated in this study (Suppl. material 1).

The two species were resolved as members of the *A. schaffneria* clade, consistent with the topology of Xu et al. (2020). Within this clade, nine well-supported subclades were recovered (Fig. 2). *Asplenium brasiliense* and *A. douglasii* formed a strongly supported monophyletic group (ML bootstrap support [MLBS] = 100; BI posterior probability [BIPP] = 1.00), with the two species being sister to one another. This *Antigramma* subclade, as designated here, was recovered as sister to the *A. varians* subclade, together forming a larger lineage within the *A. schaffneria* clade.

**Figure 1.**
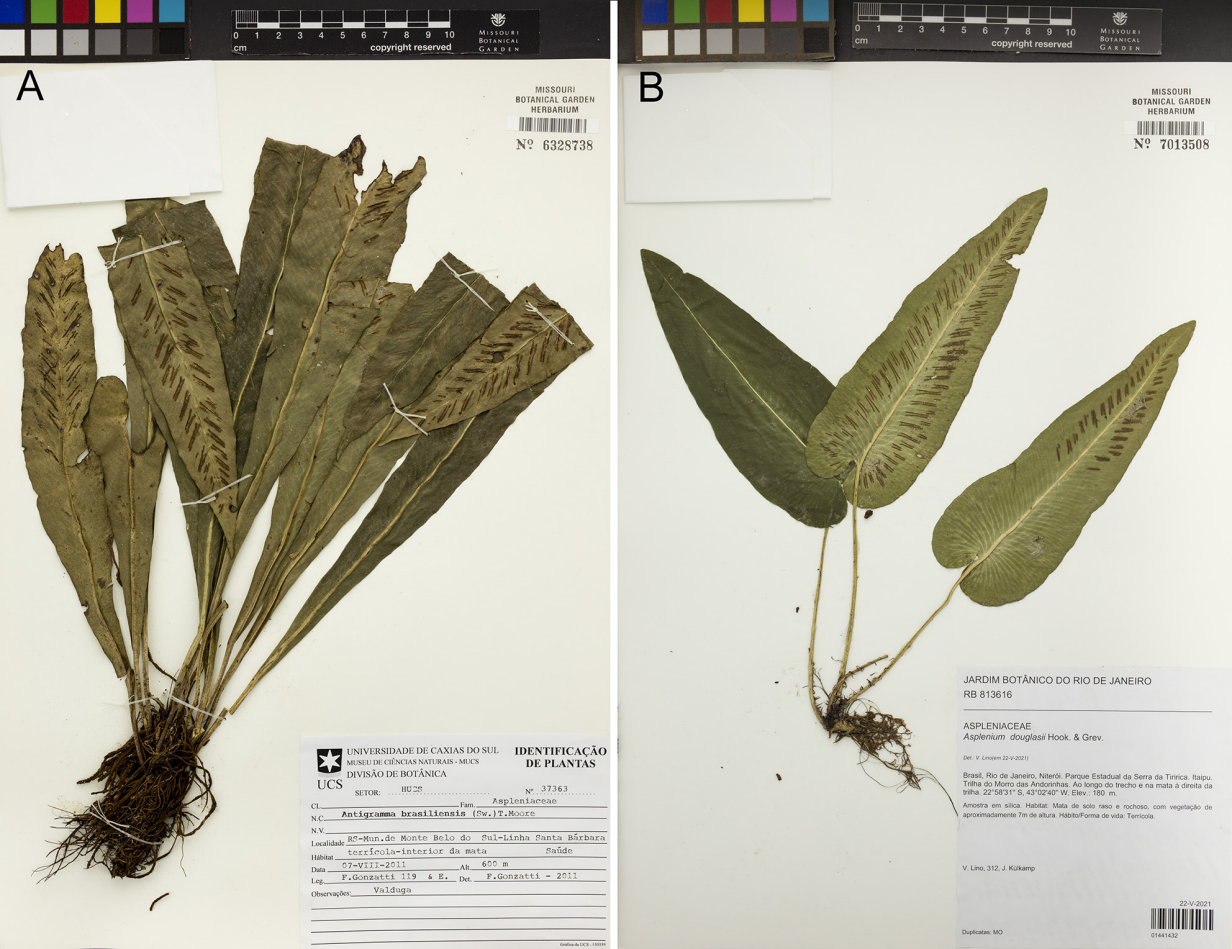
Herbarium specimens showing the morphological characteristics of two endemic South American *Asplenium* species. **A**. *Asplenium brasiliense* (*P. Gonzatti 37363*); **B**. *Asplenium douglasii* (*V. Lino 312*).

**Figure 2.**
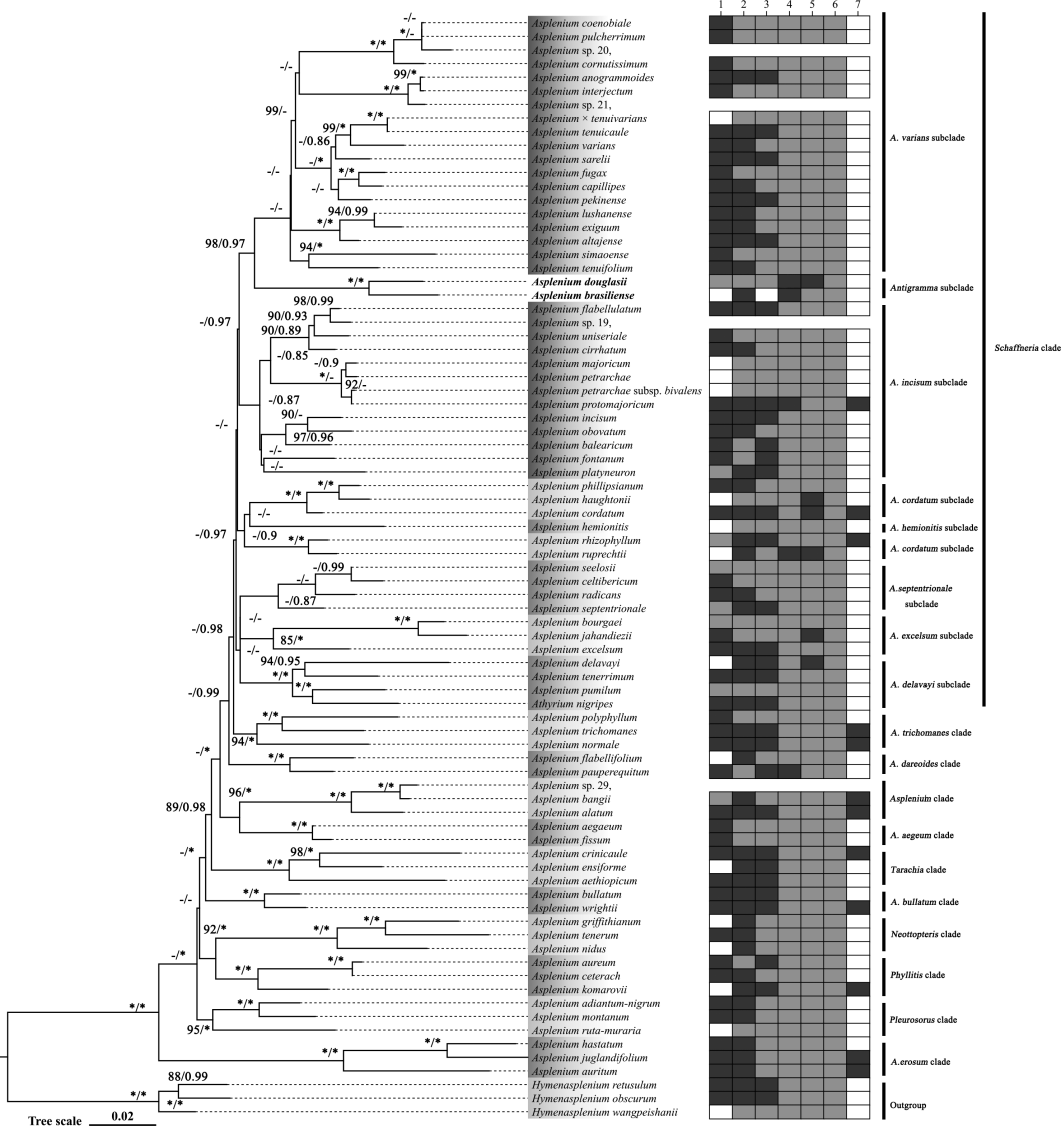
Phylogenetic position of *Asplenium brasiliense* and *A. douglasii* based on four plastid markers (*atpB*, *rbcL*, *rps4* and *rps4–trnS*, and *trnL* and *trnL–trnF*). The numbers associated with the branches indicate maximum likelihood bootstrap support (MLBS) and Bayesian inference posterior probability (BIPP). Only MLBS ≥ 80 and BIPP ≥ 0.85 are shown. An asterisk indicates MLBS = 100 and BIPP = 1.00. A dash (–) indicates that the support value is below the threshold and is therefore not shown. Morphological character codes at the tips of the tree are as follows: 1. Degree of lamina division (blank = simple or nearly simple; gray = shallowly lobed; black = clearly pinnate); 2. Overall lamina shape (blank = strap-shaped or tongue-shaped; gray = oblong or ovate; black = lanceolate); 3. Petiole-to-lamina length ratio (blank = shorter than the lamina; gray = approximately equal to the lamina; black = longer than the lamina); 4. Venation type (blank = free; gray = nearly free; black = clearly reticulate); 5. Sorus shape (blank = short linear; gray = linear; black = extremely narrow and elongated); 6. Sorus length relative to the leaf vein (blank = shorter than the vein; gray = equal to the vein; black = nearly the full length); 7. Relative development of basal auricles (near the base of the rachis) vs. apical auricles (near the leaf tip) (blank = absent; gray = occasional; black = common).

### Morphological and spore micromorphological comparisons

**Figure 3.**
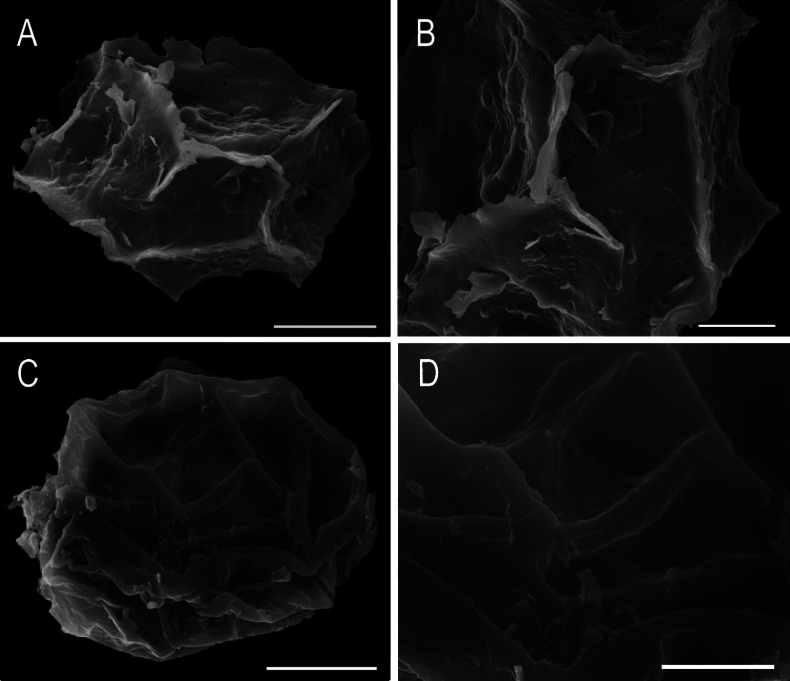
Spore ornamentation of **A, B**. *Asplenium brasiliense* and **C, D**. *A. douglasii*. Scale bars: 12.5 μm (**A**, **D**); 5 μm (**B**); 25 μm (**C**).

*Asplenium brasiliense* and *A. douglasii* are lowland species that grow terrestrially or among rocks. Both species have entire laminae, anastomosing venation with free terminations near the margin, and paired indusia fixed laterally on the major areolae and opening toward each other (Fig. 1). *Asplenium brasiliense* from Argentina, Brazil, and Paraguay can be distinguished by its lanceolate fronds, short petioles, linear sori (not extremely elongated) densely arranged near the midrib, and cristate spores (Figs 1A, 3A, 3B). However, Peruvian material with elliptic (rarely rhombiform) laminae and sori confined to the upper half of the lamina (mostly midway between the costa and margin) may represent a new species. Because molecular evidence is lacking, this material is tentatively treated as *A. brasiliense* in this study. *Asplenium douglasii* has narrowly cordiform fronds, longer petioles (equal to or longer than the lamina), narrow sori arranged between the midrib and the leaf margin, and lophate spores (Figs 1B, 3C, 3D).

## Discussion

### Phylogenetic placement of *Asplenium brasiliense* and *A. douglasii*

The two South American spleenworts, *Asplenium brasiliense* and *A. douglasii*, possess an unusual combination of morphological characters, including simple fronds, anastomosing venation, and paired indusia opening toward each other. Because of this morphological uniqueness, they have historically been transferred among several satellite genera, including *Antigramma*, *Hemidictyum*, *Phyllitis*, and *Scolopendrium* (Presl 1836; Hooker and Bauer 1842; Hooker 1846; Kunze 1850; Moore 1857; Kuntze 1891), with no consensus regarding their systematic position or phylogenetic relationships.

This study provides the first molecular phylogenetic evidence clarifying the phylogenetic positions of these two enigmatic species. The analyses, based on four chloroplast markers (*atpB*, *rbcL*, *rps4* and *rps4–trnS*, and *trnL* and *trnL–trnF*), robustly resolved both species as members of the *A. schaffneria* clade, consistent with the topology of Xu et al. (2020), as shown in Fig. 2. Within this clade, *A. brasiliense* and *A. douglasii* formed a strongly supported monophyletic group, with the two species being sister to one another. This *Antigramma* lineage, as designated here, was recovered as sister to the *A. varians* subclade, together forming a larger lineage within the *A. schaffneria* clade. The results demonstrate that, despite their striking morphological divergence from typical *Asplenium* species—which usually have free veins, unilaterally opening indusia, and more dissected fronds—*A. brasiliense* and *A. douglasii* are firmly nested within *Asplenium* s.l. This situation is analogous to that of other taxa historically recognized as separate satellite genera, such as *Ceterach* (with abaxial leaf surfaces densely covered with small scales and veins anastomosing to form hexagonal areolae near the margin) and *Ceterachopsis* (with deeply pinnatifid leaves, alternate lobes, glabrous abaxial surfaces, and free veins), both of which have also been resolved within *Asplenium* by molecular phylogenetic evidence (Xu et al. 2020). Their morphological peculiarities therefore represent specialized evolutionary derivations rather than evidence supporting generic segregation.

### Morphological transition and evolutionary significance

**Figure 4.**
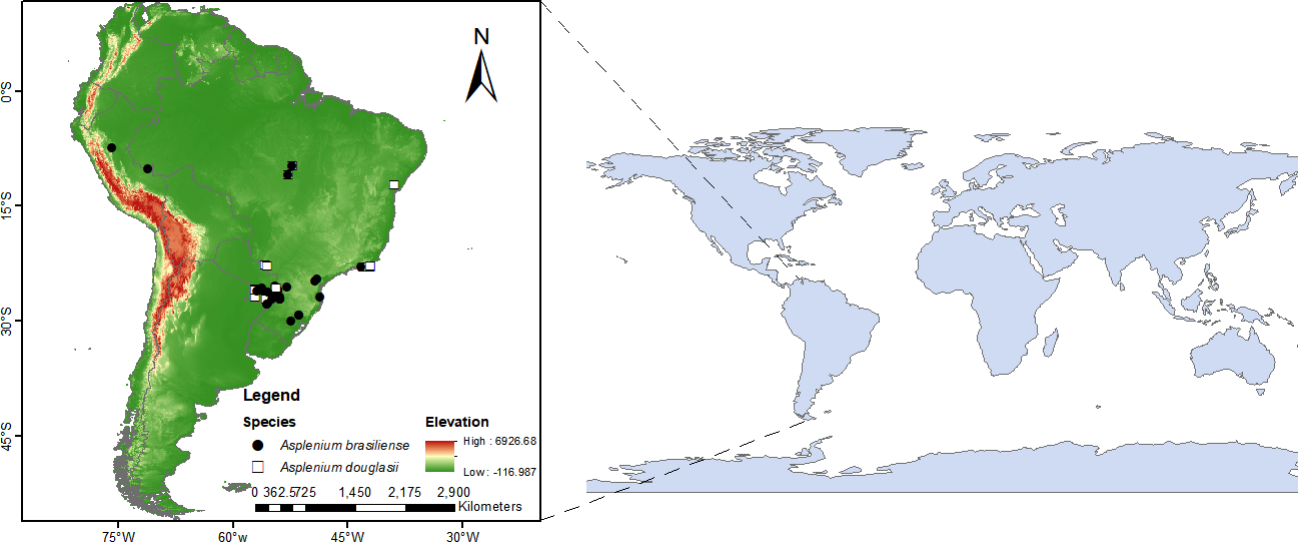
Geographic distributions of *Asplenium brasiliense* and *A. douglasii* based on examined herbarium specimens from F, FCQ, HOXA, K, LI, MA, MARY, MO, MU, NY, PI, PY, SI, UNILA, USM, UT, and Z+ZT. Black circles indicate collection localities of *A. brasiliense*, and open squares indicate collection localities of *A. douglasii*.

*Asplenium brasiliense* and *A. douglasii* belong to a highly reduced, simple-leaved lineage. Their simple fronds, anastomosing venation, and paired indusia opening toward each other on both sides of the veins distinguish them from most congeners. Nevertheless, chloroplast sequence data robustly place them within the *A. schaffneria* clade. This morphological syndrome may reflect adaptation to specific understory environments in South American forests. Simple, entire leaves with anastomosing venation are often associated with shaded, humid habitats in which light is limited and water availability is high. Reduced leaf dissection may optimize light capture under deep shade, whereas anastomosing venation may provide hydraulic redundancy against embolism. Their geographic distributions (Fig. 4, Suppl. material 2) show that both species occur in lowland forests of the Atlantic Forest and neighboring regions extending into western Amazonia, which are known for high humidity and closed canopies (Ribeiro et al. 2009).

Within the genus *Asplenium*, simple leaves with anastomosing venation are not restricted to the *A. schaffneria* clade; they also occur in other lineages, including the *A. hemionitis* subclade, which is primarily distributed in the Mediterranean and Macaronesian regions (Ormonde 1996); the *A. nidus* complex, a predominantly pantropical group extending into subtropical regions (Holttum 1974); and the *Antigramma* lineage, which is largely confined to the Neotropical region (Sylvestre and Windisch 2002). Furthermore, paired indusia opening toward each other on both sides of the veins are also found in other lineages within *Asplenium*, such as some species previously placed in the satellite genera *Phyllitis* and *Scolopendrium* (Pinter et al. 2002; Vasheka et al. 2016). This indicates that these characters have evolved independently multiple times across the genus (Xu et al. 2020; Fig. 2). The results suggest that the *Antigramma* lineage is not closely related to these other groups possessing simple, anastomosing-veined leaves or paired indusia opening toward each other. This parallel evolution of simple fronds from pinnate ancestors and paired indusia from solitary indusia underscores the high developmental lability of leaf dissection in ferns.

## Additional information

### Artificial Intelligence (AI) use

The authors accept full responsibility for the content of the manuscript, including the disclosure of any use of AI.

No AI tools were used in the preparation of this manuscript.

### Author ORCIDs

L.-B. Zhang https://orcid.org/0000-0002-4905-040X

K.-W. Xu https://orcid.org/0000-0003-2239-0487

### Data availability

All of the data that support the findings of this study are available in the main text or Supplementary Information.

## Supplementary materials

Supplementary material 1 List of voucher specimens and GenBank accession numbers used in the phylogenetic analyses Data type xlsx Explanation note A dash (–) indicates missing data. https://binary.pensoft.net/file/1724905This dataset is made available under the Open Database License (http://opendatacommons.org/licenses/odbl/1.0/). The Open Database License (ODbL) is a license agreement intended to allow users to freely share, modify, and use this Dataset while maintaining this same freedom for others, provided that the original source and author(s) are credited. Jing-Shuai Zhang, Ming-Hui Du, Li-Bing Zhang, Ke-Wang Xu

Supplementary material 2 Voucher specimens used to generate the distribution map (Fig. 4) Data type xlsx https://binary.pensoft.net/file/1724906This dataset is made available under the Open Database License (http://opendatacommons.org/licenses/odbl/1.0/). The Open Database License (ODbL) is a license agreement intended to allow users to freely share, modify, and use this Dataset while maintaining this same freedom for others, provided that the original source and author(s) are credited. Jing-Shuai Zhang, Ming-Hui Du, Li-Bing Zhang, Ke-Wang Xu
